# Evaluation of Immune Dysregulation in an Austrian Patient Cohort Suffering from Myalgic Encephalomyelitis/Chronic Fatigue Syndrome

**DOI:** 10.3390/biom11091359

**Published:** 2021-09-14

**Authors:** Lena Lutz, Johanna Rohrhofer, Sonja Zehetmayer, Michael Stingl, Eva Untersmayr

**Affiliations:** 1 Institute of Pathophysiology and Allergy Research, Center for Pathophysiology, Infectiology and Immunology, Medical University of Vienna, 1090 Vienna, Austria; n1547777@students.meduniwien.ac.at (L.L.); johanna.rohrhofer@meduniwien.ac.at (J.R.); 2 Institute of Medical Statistics, Center for Medical Statistics, Informatics and Intelligent Systems, Medical University of Vienna, 1090 Vienna, Austria; sonja.zehetmayer@meduniwien.ac.at; 3 Facharztzentrum Votivpark, 1090 Vienna, Austria; ordination@neurostingl.at

**Keywords:** myalgic encephalomyelitis/chronic fatigue syndrome, immunodeficiency, immune dysfunction, immune activation, inflammation

## Abstract

Myalgic encephalomyelitis/chronic fatigue syndrome (ME/CFS) is a severe multi-systemic disease characterized by debilitating fatigue that is not relieved by rest. The causes of the disease are still largely unexplained, and no causative treatment is currently available. Changes in the immune response are considered as fundamental in the development of ME/CFS. Thus, we aimed to evaluate the immunological profile of ME/CFS patients in a retrospective data analysis. As part of the routine workup for ME/CFS patients, a differential blood count, leukocyte subtyping, and quantification of immunoglobulins and IgG subclasses, as well as a complement analysis, was performed. Out of 262 ME/CFS patients, 64.9% had a reduction or deficiency in at least one of the listed immune parameters. In contrast, 26.3% showed signs of immune activation or inflammation. A total of 17.6% of the ME/CFS patients had an unclassified antibody deficiency, with IgG3 and IgG4 subclass deficiencies as the most common phenotypes. Reduced MBL (mannose-binding lectin) levels were found in 32% of ME/CFS patients, and MBL deficiency in 7%. In summary, the present results confirmed the relevance of immune dysfunction in ME/CFS patients underlining the involvement of a dysfunctional immune response in the disease. Thus, immune parameters are relevant disease biomarkers, which might lead to targeted therapeutic approaches in the future.

## 1. Introduction

Myalgic encephalomyelitis/chronic fatigue syndrome (ME/CFS) is a multi-systemic disease with an estimated prevalence of 0.3 to 0.8% in the general population [1] Approximately 25,000 patients of all age and socioeconomic groups are calculated to be affected in Austria (as of January 2019 [2]), but precise data is missing. Women are affected twice as often as men [3]. ME/CFS is associated with an enormous disease burden and can lead to complete incapacity to work. The onset is usually acute with flu-like symptoms but can also manifest in a subacute or insidious manner. The disease is defined by chronic debilitating fatigue lasting more than six months and various other symptoms such as sleep disturbances, mental and physical pain, neurological and cognitive impairment, as well as autoimmunity or immunodeficiencies [4]. Rest does not relieve the fatigue, which is typically worsened after physical and mental exertion (post-exertional malaise, PEM). The PEM is essential to distinguish ME/CFS from other diseases, in which patients feel better after exertion, such as depressive disorders [5].

ME/CFS is classified as a neurological disease with G93.3 in the International Classification of Diseases (ICD) by the World Health Organization (WHO). Originally, the disease was coined as ME. As limited evidence for brain inflammation was found, the term CFS is now more preferred in the scientific community [6]. Currently there are no biological markers for the disease. Therefore, diagnosis depends on case definition and exclusion of other diseases [4]. Due to the lack of biomarkers for appropriate diagnosis, the distress of ME/CFS patients is often very high. Up to 90% of affected patients are not properly diagnosed, but prematurely labelled as psychosomatic [5]. Furthermore, currently there are no causative treatment options available. Studies suggest that some ME/CFS patients recover over time, but most remain with disabilities for several years [7].

Despite numerous studies, the underlying pathomechanisms of ME/CFS are poorly understood. Some studies suggest single causes, while most studies underline the multifactorial nature of the disease. A dysregulation of the immune system or the autonomic nervous system, as well as metabolic disturbances, genetic predisposition and environmental influences may contribute to this complex disease [6,8,9].

Infectious diseases have been repeatedly postulated as a potential trigger of ME/CFS [10]. In about 50% of cases, an acute viral infection seems to trigger ME/CFS, resulting in a complex cascade of immune disturbances, which might contribute to the onset of symptoms [11]. Numerous viruses have been discussed to be associated with ME/CFS, including enteroviruses, herpes viruses (especially Epstein–Barr virus, EBV), retroviruses, parvovirus B19, hepatitis C virus, and Ross River virus (RRV) [11,12]. After SARS-CoV-2 infections, a subgroup of patients met the diagnostic criteria for ME/CFS six months after the acute viral infection [8]. The virulence of the pathogens alone cannot explain the onset of ME/CFS, which might be rather linked to an abnormal response to the infection itself [13]. ME/CFS patients show various symptoms of immune dysfunction [5,14]. Immunologic changes commonly reported are increased T-cell activation, a biased type 1/type 2 immune response, altered cytokine secretion, altered immunoglobulin levels, natural killer cell dysfunction, or increased complement activation products [15,16]. ME/CFS also shares certain features with autoimmune diseases. Both diseases are more common in women and are characterized by increased inflammation. Moreover, autoantibodies against the β_2_ adrenergic receptor (β_2_ AdR) and M3 muscarinic receptor were detected in ME/CFS patients [17].

As changes in the immune response are considered to play a key role in the development of ME/CFS, we aimed to evaluate the immunological profile of ME/CFS patients in a retrospective manner. The primary objective of this retrospective study was to analyse the frequency of immune dysfunction in a cohort of Austrian ME/CFS patients for a better understanding of the pathophysiology of ME/CFS.

## 2. Materials and Methods

A retrospective data analysis was conducted on medical data of ME/CFS patients treated during the study period March 2019 to August 2020. ME/CFS was diagnosed by a specialized neurologist based on exclusion of other medical conditions associated with profound fatigue and based the IOM criteria for the diagnosis G93.3 ME/CFS. Three symptoms and at least one of two additional manifestations were required for diagnosis of ME/CFS. The main symptoms were (1) a substantial reduction or impairment in the ability to engage in pre-illness activity levels for more than six months, which is accompanied by fatigue; (2) post-exertional malaise (PEM); and (3) unrefreshing sleep. Additional manifestations were cognitive impairment and/or orthostatic intolerance [18]. In case of a suggestive history, immunodiagnostics were performed as part of the routine workup for ME/CFS patients including a differential blood count, leukocyte subtyping, immunoglobulins and IgG subclasses, as well as a complement analysis. During the 18-month study period, 351 ME/CFS patients over 18 years of age were followed-up. Eighty-nine patients were excluded from further assessments as immunodiagnostic results were not fully available. The final evaluation was based on immunodiagnostic data of 262 ME/CFS patients independent of gender distribution. All values were compared to defined laboratory norm levels (Supplementary Table S1). The collected data was analysed using descriptive statistics (absolute and relative frequencies) and 95% Clopper–Pearson confidence intervals (95%CI) for the number of patients with reduced and/or elevated laboratory values to assess relevant parameters for the ME/CFS compared to the norm laboratory reference values within the general population. To statistically compare the presence of immune changes in male versus female ME/CFS patients and in two different age groups (18–40 and 41–80) for each parameter, frequencies were statistically compared by a Fisher exact test (due to small group frequencies). The significance level was set to 0.05, however, due to the exploratory character of the analysis, *p*-values were interpreted descriptively and no adjustment for multiplicity was performed. The study was approved by the Ethics Committee of the Medical University of Vienna Wien (EK No. 1441/2020), in accordance with the 1964 Declaration of Helsinki and its later amendments.

## 3. Results

### 3.1. Demographic Patient Data

A specialized neurologist diagnosed ME/CFS based on the exclusion of other medical conditions associated with profound fatigue and the IOM criteria for the diagnosis G93.3 ME/CFS. Out of the total number of 262 patients, 207 patients met all IOM criteria for ME/CFS diagnosis. Fifty-five patients had chronic fatigue, which either did not last for more than six months at the time point of evaluation or a clear symptom onset could not be defined. Twice as many women suffered from ME/CFS (179 female, 83 male patients). The mean age of patients was 41 years (18–79 years). The average duration of disease until immune evaluation was 9.4 years, with a range from 1–39 years. A history of frequent recurrent infections was reported in 60% of all patients, including 45 patients (17.2%) who could recall having actively undergone EBV infection, also termed mononucleosis. Out of the total 262 patients, there were 194 (74%) patients who had positive EBV antibodies (either IgG or IgM). Table 1 summarizes the demographic characteristics of the study population.

### 3.2. Immune Parameters in ME/CFS Patients

**Figure 1:**
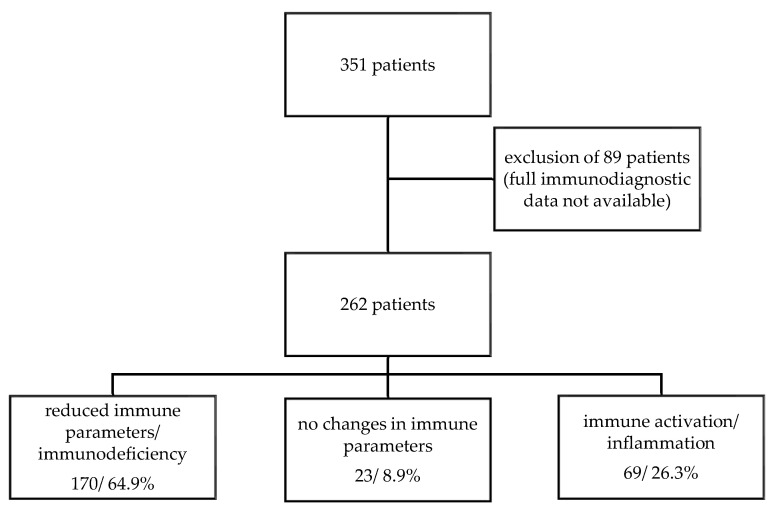
Patient flow chart.

Out of the total number of 262 ME/CFS patients, 170 (64.9%) have a reduction or deficiency in at least one of the listed immune parameters. In contrast, 69 of the patients (26.3%) showed signs of immune activation or inflammation, characterized by the increase of one of the evaluated immune parameters (Figure 1).

### 3.3. Reduced Humoral Immune Response in ME/CFS Patients

**Figure 2:**
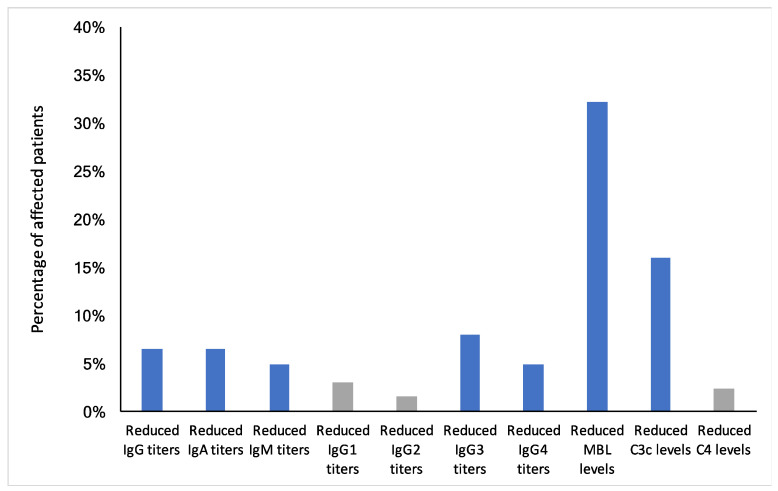
Frequency of reduced humoral immune parameters in 262 ME/CFS patients. Reduced humoral immune parameters not found within the norm reference range as indicated by the 95% Clopper–Pearson confidence interval calculation are marked as blue bars. Gray bars indicate parameters, which lie within the 95% CI of the general population.

To evaluate humoral immune parameters in ME/CFS patients, immunoglobulins IgM, IgA, IgG, and IgG subclasses, as well as MBL (mannose-binding lectin), C3c, and C4 levels, were measured and compared to norm laboratory values. IgA, as well as total IgG levels, were decreased in 6.5% of all patients. IgM levels were below the norm values in 4.9% of ME/CFS patients. The most prominent reduction of the IgG subclasses was found for IgG3 in 8% of the patients and for IgG4 in 4.9% of the patients. Reduced MBL levels were detected in 32.1% of all patients, and reduced C3c levels in 16% of all patients (Figure 2).

Using the calculated 95% Clopper–Pearson confidence interval, we aimed to define the reduced humoral immune parameters not found within the norm reference range and, thus, have found a greater deviation than the 2.5% (7 out of 262 subjects) expected within the general population. For reduced IgG, IgA, IgM, IgG3, IgG4, MBL and C3c levels, we observed above-average deviations indicating specific relevance for ME/CFS patients (marked as blue bars in Figure 2 and Supplementary Table S2).

For each immune parameter, we additionally compared male patients (n = 83) with female patients (n = 179) and patients from two different age groups (18–40 years (n = 131) vs. 41–80 years (n = 131)), with a Fisher exact test, to see if there were significant differences in the frequencies of reduced immune parameters between the groups. Except for reduced IgM (*p*-value, 0.02) and IgG2 (*p*-value, 0.01) levels between male and female patients, we observed no major differences (Supplementary Tables S3 and S4). Reduced immune parameters are not particularly clustered in any of the ME/CFS patient groups, but are evenly distributed between male and female patients and within the two different age groups.

**Figure 3:**
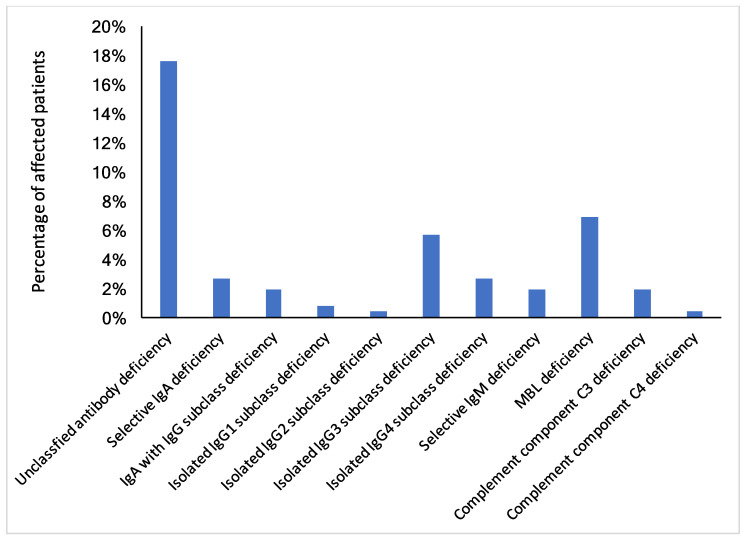
Frequency of diagnosed immunodeficiencies in 262 Austrian ME/CFS patients based on laboratory testing and clinical history. Blue bars indicate the percentage of clinically diagnosed immunodeficiencies in the ME/CFS patients’ cohort. Due to the descriptive evaluation of the data, no statistical comparison has been performed.

To define the level of immunological dysfunction, data was analysed according to available definitions for immunodeficiencies (European Society for Immunodeficiencies, ESID [19]). In addition to reduced values of the immune parameters, the presence of recurrent and/or severe infections played an important role in the evaluation and was included in the data assessment from the patients’ medical history. We observed an unclassified antibody deficiency in 17.6% of all patients. On the level of immunoglobulins and IgG subclasses, the highest percentage was found for an isolated IgG3 subclass deficiency found in 5.7% of all patients. In addition, 2.7% of patients suffered from an isolated IgG4 subclass deficiency and 2.7% of all patients were diagnosed with selective IgA deficiency. MBL deficiency was diagnosed in 6.9% of all patients (Figure 3).

With a Fisher exact test, we again compared male patients (n = 53) with female patients (n = 114) and patients from two different age groups (18–40 years (n = 131) vs. 41–80 years (n = 131)) for each parameter to detect sex- or age-related frequency clusters of immunodeficiencies. Only for isolated IgG4 subclass deficiency (*p*-value, 0.01) was a significant difference between the two age groups detected (Supplementary Tables S5 and S6). All other immunodeficiencies seemed to be evenly distributed across ME/CFS patients regardless of sex and age.

### 3.4. Combination of Reduced Cellular and Humoral Immune Response in ME/CFS Patients

In addition to humoral immune parameter reductions and immunodeficiencies, we also integrated changes on cellular levels in our evaluations. Table 2 gives an overview regarding combinations of immunoglobulin reduction or deficiencies with different immune cell types. The largest number of patients (n = 8) revealed immunoglobulin reduction with reduced CD3-CD16+CD56+ NK (natural killer) cell counts. When considering the data based on an unclassified antibody deficiency, this was also the most common combination of humoral and cellular immune parameter reduction (n = 6). None of the patients with reduced antibody levels showed a decrease in CD3+CD16+CD56+ NKT cell counts.

### 3.5. Elevated Immune Parameters in ME/CFS Patients

We were additionally interested in an increase in both cellular and humoral immune parameters correlating ME/CFS with immune activation or inflammation (Table 3). Elevated levels of CD8-CD57+ NK cells were found in 23 patients and higher levels of CD4+ T-cells were detected in 12 patients. Only four patients had an increase in CD8+ T-cells. When evaluating humoral immune parameters, an increase was found mainly in IgG2 (n = 13). Elevated levels of complement parameters were rarely observed; C3 elevation was observed in one patient and C4 in three patients.

Using the calculated 95% Clopper–Pearson confidence interval, we aimed to illustrate the evaluated humoral and cellular immune parameters not found within the norm reference range and, thus, have a greater deviation than the 2.5% (7 of 262 subjects) expected within the general population. It was only for elevated CD8-CD57 + NK cell counts and elevated IgG2 antibody titers that we observed above-average deviations, indicating specific relevance for ME/CFS patients (Table 3).

## 4. Discussion

ME/CFS is a multi-systemic severe disease that might even lead to complete incapacity to work. It is characterized by chronic debilitating fatigue lasting more than six months and various other symptoms such as sleep disturbances, pain, orthostatic intolerance, neurological and cognitive changes, motor impairments and an altered immune response. For ME/CFS onset, an infection is frequently reported, and many patients suffer from recurrent viral or bacterial infections. In our study, 74% of the patients had positive EBV specific antibodies (either IgG or IgM). We are fully aware that 90% of adults are considered to have been infected with EBV [20]. However, current literature indicates that the seropositivity to EBV antibodies is decreasing in the general population and depends on the age of the evaluated populations [21,22]. Of interest, a group of 33,654 healthy subjects within the same age ranges as our patients (mean age 42.0 ± standard deviation of 23.8) showed seropositivity in 88.3% of subjects [21], which is higher than for our study populations. Moreover, patients with chronic infection and immunosuppressed patients were reported to remain negative for EBNA-1 IgG or to have only low levels in previous research [23].

Previous studies have highlighted the link between immune dysfunction and ME/CFS development [6,8,9,15,16,24]. These previous results are confirmed in a comprehensive immune evaluation of 262 ME/CFS patients, revealing mostly a reduction or even immunodeficiency in over 64% of all patients.

Immunoglobulin deficiency was a frequent diagnosis in our study. More than 17% of the ME/CFS patients had an unclassified antibody deficiency, being defined as recurrent or severe bacterial infection or autoimmune phenomena, and a deficiency of IgG, IgG subclass, IgM, IgA and/or specific antibodies, alone or in combination [19].

Previous studies show that the levels of serum IgG and the IgG subclasses appear to be reduced in some ME/CFS patients [14,16,25]. In our study, patients showed mostly a reduction of IgG3, followed by IgG4. IgG1 and IgG2 deficiencies were less frequent. These findings are in line with previous reports revealing IgG3 deficiency to be the most frequent in ME/CFS patients, with 64% of ME/CFS patients having decreased IgG3 titers [26]. In another patient cohort, single or concomitant IgG3 or IgG4 deficiency was found in 8.6% or 9% of all patients (IgG3: n = 25; IgG4: n = 26). IgG3 antibodies recognize bacterial proteins and are relevant virus-neutralizing immunoglobulins. A deficiency is therefore mainly noticeable by recurrent upper respiratory tract infections, bronchial asthma and diarrhea [16]. IgG4, which accounts for the smallest proportion of total IgG (4–6%), is referred to as an immunoregulatory antibody. Although less is known regarding its clinical significance, deficiency was suggested to be associated with autoimmunity [16,27].

In our patient cohort, 6.5% of ME/CFS patients had reduced IgA levels (<7 mg/dL) and 2.7% even had selective IgA deficiency. Selective IgA deficiency is a rare immunodeficiency with various causative triggers. In the European Union, the prevalence is estimated to be 0.001% [28]. To the best of our knowledge, there is no study which has investigated an association between IgA deficiency and ME/CFS. Thus, further studies are urgently needed to uncover a causal relationship between ME/CFS and IgA deficiency.

In our patient cohort, increased levels of at least one immunoglobulin class or subclass were found in 36 of the 262 ME/CFS Patients (13.7%). The most common increase was observed for IgG2 in 13 patients, followed by an increase in IgM in 8 patients. These results are in accordance with previous studies, demonstrating a prominent role of elevated IgM and IgG2 titers in ME/CFS [16,29]. Of interest, increased IgM titers are found in chronic EBV infections, as well as in particular autoimmune diseases like hepatitis and primary biliary cirrhosis. For a subgroup of these patients, fatigue and autonomic dysfunction, similar to ME/CFS, is a frequently observed symptom [30,31,32]. Elevated IgG, mostly of the IgG1 and IgG3 subclasses, but also IgG2, has been reported in various autoimmune diseases [33]. Thus, immune activation seems to be of special relevance in ME/CFS, as autoimmunity is discussed as causative for disease development. A recent study described an increase of autoantibodies against the β_2_ adrenergic receptor (β_2_ AdR) and M3 muscarinic receptor, both essential receptors in vasodilation in ME/CFS patients [34].

Another remarkable finding in our study is the high frequency of MBL deficiency. In our study, the cut-off level for MBL was defined at <50 ng/mL. Reduced MBL levels were seen in 32.1% of ME/CFS patients, representing the most frequently reduced immune parameter. MBL deficiency, being defined as MBL levels below 50 ng/mL, in combination with severe or recurrent infections, was found in 7%. This highlights the high frequency of MBL deficiency in ME/CFS patients, as MBL deficiency with a cut off value < 100 ng/mL is assumed to be found in 4% of the Caucasian population [16]. Up to this point, the data on MBL deficiency in ME/CFS patients is still limited. A previous study using a cut-off value <100 ng/mL for MBL deficiency found that 15% (n = 43 of 293) of ME/CFS patients were affected by this immunodeficiency. In general, ME/CFS patients had lower MBL levels than healthy controls, and more than half reported an increased susceptibility to upper and lower respiratory tract infections [16]. The role of the complement system is discussed in the context of ME/CFS. The complement system comprises several proteins that interact with each other via complex regulatory mechanisms and play a role in the amplification of the immune response, the lysis of pathogens, and even in the maturation of synapses and angiogenesis [35]. Sixteen percent of our ME/CFS patients had reduced C3c values, representing the most frequently reduced immune parameter after reduced MBL levels. Very little information is currently available on changes in complement factor levels in ME/CFS patients. In ME/CFS patients exposed to physical stress, significantly increased levels of C4a were found in comparison to healthy individuals [35]. In our study we identified three patients with elevated values of C4.

Several studies have reported alterations of various cellular compartments in ME/CFS patients with a decreased NK cell function and a shift towards a T-helper-2-type immune response [15,36,37]. IL-4, the marker cytokine of T-helper-2 (Th2) cells plays an important role in allergy-associated inflammatory responses, as it particularly stimulates mast cells and other effector cells to proliferate. In addition, other mediators, such as prostaglandins and chemokines, which are strongly produced in the context of allergic inflammation, can stimulate the selective recruitment of Th2 cells [38]. Regarding the total number of CD4+ T helper cells, a few studies have reported a decrease in the number of cells, whereas the majority did not show any differences. In our study, we were not only interested in the individual humoral immune parameter reductions and deficiencies in ME/CFS patients, but also in their combinations with changes in cellular levels. The combination that was recorded for the largest number of patients (n = 8) was immunoglobulin reduction with reduced CD3-CD16+CD56+ NK cell counts. When considering the data based on an unclassified antibody deficiency, this was also the most common combination (n = 6). In a study from Tirelli et al., a substantial reduction in CD3-CD16+CD56+ cells was reported for ME/CFS patients, while no significant differences were found in the absolute numbers of circulating total T cells (CD3+) and of total helper CD4+ or cytotoxic CD8+ T cells [39].

Looking at the distribution of the different immune parameters among male versus female ME/CFS patients and in two different age groups, we observed hardly any significant differences, suggesting that the immune alterations are independent of sex and age influences.

We are fully aware regarding the limitations of our study. We have performed a retrospective analysis of patients’ data. This limitation is overcome by the fact that for most patients, extensive medical history and several laboratory evaluations were available. Moreover, due to the chosen study design and the lack of further functional analysis, we cannot distinguish between primary and secondary immunodeficiencies in this patient cohort. From a statistical point of view the study population is rather small, which might not allow discrimination of all the relevant parameters for ME/CFS patients compared to values determined for the general population. Thus, larger studies in ME/CFS patients are urgently needed in search for disease relevant biomarkers.

In summary, the present results confirm the relevance of immune dysfunction in ME/CFS patients underlining the involvement of a dysfunctional immune response in the disease. Thus, immune parameters are relevant biomarkers in ME/CFS patients to identify patients with potential responses to immune-modulating treatment for future targeted therapy of ME/CFS.

## Figures and Tables

**Table 1:** Demographic and clinical characteristics of the study population (n = 262).

Parameter	Absolute Frequency	Mean	Range
Sex (male/female)	83/179		
Age (years)		41	18–79
Age groups (18–40 years/41–80 years)	131/131		
Fulfilling of all IOM-criteria for the diagnosis ME/CFS	207		
Duration of disease until immunodiagnostics (years)		9.4	1–39
Patients with recurrent infections	158		
Patients diagnosed with acute EBV infection at onset of fatigue	45		
positive EBV antibodies	194		

**Table 2:** Number of patients with humoral and cellular immune parameter reductions.

Humoral Parameter	Cellular Parameter	No. of Patients (95%CI) (n = 262)
	CD4+ T-cell lymphopenia	5 (2–12)
	reduced CD8-CD57+ NK cell counts	6 (2–13)
Reduction of at least one of total IgG, IgG1, IgG2, IgG3, IgA or IgM levels in combination with…	reduced CD3+CD16+CD56+ NKT cell counts	0 (0–4)
	reduced CD3-CD16+CD56+ NK cell counts	8 (3–16)
	CD4+ T-cell lymphopenia	4 (1–10)
	CD8+ T-cell lymphopenia	3 (1–9)
Unclassified antibody deficiency in combination with…	reduced CD8-CD57+ NK cell counts	4 (1–10)
	reduced CD3+CD16+CD56+ NKT cell counts	0 (0–4)
	reduced CD3-CD16+CD56+ NK cell counts	6 (2–13)
	CD4+ T-cell lymphopenia	4 (1–10)

**Table 3:** Number of ME/CFS patients with elevated immune parameters (humoral and cellular).

Parameter	No. of Patients (95%CI) (n = 262)
CD4+ T-cells	12 (6–21)
CD8+ T-cells	4 (1–10)
**CD8-CD57+ NK cell counts**	**23 (15–34)**
CD3+CD16+CD56+ NKT cell counts	7 (3–14)
CD3-CD16+CD56+ NK cell counts	4 (1–10)
IgG	4 (1–10)
IgA	4 (1–10)
IgM	8 (3–16)
IgG1	5 (2–12)
**IgG2**	**13 (7–22)**
IgG3	3 (1–9)
IgG4	5 (2–12)
C3	1 (0–6)
C4	3 (1–9)

## Data Availability

The data presented in this study are available in the article and supplementary material.

## Supplementary Materials

The following are available online at https://www.mdpi.com/article/10.3390/biom11091359/s1, Table S1: Norm values used for laboratory diagnosis, Table S2: Number of ME/CFS patients with reduced humoral immune parameters, Table S3: Differences of reduced immune parameters in male versus female ME/CFS patients, Table S4: Differences of reduced immune parameters in two age groups of ME/CFS patients, Table S5: Immunodeficiencies in male versus female ME/CFS patients, Table S6: Immunodeficiencies in different age groups of ME/CFS patients.
