# Supplementary material for: Evaluation of Immune Dysregulation in an Austrian Patient Cohort Suffering from Myalgic Encephalomyelitis/Chronic Fatigue Syndrome
Source: Biomolecules. 2021 Sep 14;11(9):1359. doi: 10.3390/biom11091359 (PMC8465819; doi:10.3390/biom11091359)
Supplement: Supplementary file 1 [file biomolecules-11-01359-s001.zip › biomolecules-1292283-supplementary.pdf]

biomolecules

MDPI

Supplementary information

Supplementary table S1. Norm values used for laboratory diagnosis.

Immune Parameter	Reference Value
CD4+ T cells	500-1500 cells/µl
CD8+ T cells	250-1000 cells/µl
CD8-CD57+ NK cells	30-180 cells/µl
CD3+16+56+ NKT cells	20-220 cells/µl
CD3-CD16+CD56+ NK cells	90-600 cells/µl
IgG	7-16 g/l
IgA	0.7-2.3 g/l
IgM	0.4-2.3 g/l
IgG1	2.8-8.0g/l
IgG2	1.15-5.70g/l
IgG3	0.24-1.25g/l
IgG4	0.052-1.25g/l
MBL	<300ng/ml
C3c	0.9-1.8 g/l
C4	0.1-0.4 g/l

Supplementary table S2. Number of ME/CFS patients with reduced humoral immune parameters

Parameter	No. of patients (95%CI) (n= 262)
**reduced IgG titers**	**17 (10-27)**
**reduced IgA titers**	**17 (10-27)**
**reduced IgM titers**	**13 (7-22)**
reduced IgG1 titers	8 (3-16)
reduced IgG2 titers	4 (1-10)
**reduced IgG3 titers**	**21 (13-31)**
**reduced IgG4 titers**	**13 (7-22)**
**reduced MBL levels**	**84 (69-100)**
**reduced C3c levels**	**41 (30-54)**
reduced C4 levels	6 (2-13)

Supplementary table S3. Differences of reduced immune parameters in male versus female ME/CFS patients

parameter	male (n= 83)	female (n=179)	p-value
reduced IgG titers	5 (6.0 %)	12 (6.7%)	1
reduced IgA titers	3 (3.6%)	14 (7.8%)	0.28
**reduced IgM titers**	**8 (9.6%)**	**5 (2.8%)**	**0.02**
reduced IgG1 titers	1 (1.2%)	7 (3.9%)	0.44
**reduced IgG2 titers**	**4 (4.8%)**	**0**	**0.01**
reduced IgG3 titers	10 (12.0%)	11 (6.1%)	0.14
reduced IgG4 titers	2 (2.4%)	11 (6.1%)	0.24
reduced MBL levels	30 (36.1%)	54 (30.2%)	0.39
reduced C3c levels	10 (12.0%)	31 (17.3%)	0.36

reduced C4 levels	1 (1.2%)	5 (2.8%)	0.67
no immune changes	35 (42.2%)	76 (42.5%)	1

For each parameter, frequencies were statistically compared by a Fisher exact test.

Supplementary table S4. Differences of reduced immune parameters in two age groups of ME/CFS patients

parameter	18-40 years (n= 131)	41-80 years (n= 131)	p-value
reduced IgG titers	6 (4.6%)	11 (8.4%)	0.32
reduced IgA titers	5 (3.8%)	12 (9.2%)	0.13
reduced IgM titers	6 (4.6%)	7 (5.3%)	1
reduced IgG1 titers	2 (1.5%)	6 (4.6%)	0.28
reduced IgG2 titers	4 (3.1%)	0	0.12
reduced IgG3 titers	8 (6.1%)	13 (9.9%)	0.36
reduced IgG4 titers	9 (6.9%)	4 (3.1%)	0.25
reduced MBL levels	38 (29%)	46 (35.1%)	0.35
reduced C3c levels	26 (19.8%)	15 (11.5%)	0.09
reduced C4 levels	3 (2.3%)	3 (2.3%)	1
no immune changes	61 (46.6%)	50 (38.2%)	0.21

For each parameter, frequencies were statistically compared by a Fisher exact test.

Supplementary table S5. Immunodeficiencies in male versus female ME/CFS patients

Parameter	Male (n= 53)	Female (n=114)	p-value
unclassfied antibody deficiency	14 (26.4%)	32 (28.1%)	0.85
selective IgA deficiency	0	7 (6.1%)	0.1
IgA with IgG subclass deficiency	1 (1.9%)	4 (3.5%)	1
isolated IgG1 subclass deficiency	0	2 (1.8%)	1
isolated IgG2 subclass deficiency	1 (1.9%)	0	0.32
isolated IgG3 subclass deficiency	6 (11.3%)	9 (7.9%)	0.56
isolated IgG4 subclass deficiency	0	7 (6.1%)	0.1
selective IgM deficiency	3 (5.7%)	2 (1.8%)	0.33
MBL deficiency	7 (13.2%)	11 (9.6%)	0.59
complement component C3 deficiency	0	5 (4.4%)	0.18
complement component C4 deficiency	0	1 (0.9%)	1
no immune changes	22 (41.5%)	39	0.39

For each parameter, frequencies were statistically compared by a Fisher exact test.

Supplementary table S6. Immunodeficiencies in different age groups of ME/CFS patients

Parameter	18-40 years (n= 86)	41-80 years (n= 81)	p-value
unclassfied antibody deficiency	23 (26.7%)	23 (28.4%)	0.86
selective IgA deficiency	2 (2.3%)	5 (6.2%)	0.27
IgA with IgG subclass deficiency	2 (2.3%)	3 (3.7%)	0.67
isolated IgG1 subclass deficiency	2 (2.3%)	0	0.5
isolated IgG2 subclass deficiency	1 (1.2%)	0	1
isolated IgG3 subclass deficiency	6 (6.9%)	9 (11.1%)	0.42
**isolated IgG4 subclass deficiency**	**7 (8.1%)**	**0**	**0.01**
selective IgM deficiency	3 (3.5%)	2 (2.5%)	1
MBL deficiency	9 (10.5%)	9 (11.1%)	1
complement component C3 deficiency	2 (2.3%)	3 (3.7%)	0.67
complement component C4 deficiency	1 (1.2%)	0	1
No immune changes	30 (34.9%)	31 (38.3%)	0.75

For each parameter, frequencies were statistically compared by a Fisher exact test.
